# Exploring Metabolic Consequences of *CPS1* and *CAD* Dysregulation in Hepatocellular Carcinoma by Network Reconstruction

**DOI:** 10.2147/JHC.S239039

**Published:** 2020-01-07

**Authors:** Ozbil E Dumenci, Abellona MR U, Shahid A Khan, Elaine Holmes, Simon D Taylor-Robinson

**Affiliations:** 1 Imperial College London, London, UK

**Keywords:** metabonomics, reprogramming, bioinformatics, liquid biopsy, hepatocytes

## Abstract

**Purpose:**

Hepatocellular carcinoma (HCC) is the fourth commonest cause of cancer-related mortality; it is associated with various genetic alterations, some involved in metabolic reprogramming. This study aimed to explore the potential metabolic impact of *Carbamoyl Phosphate Synthase I* (*CPS1*) and *carbamoyl phosphate synthetase/aspartate transcarbamoylase/dihydroorotase (CAD)* dysregulation through the reconstruction of a network that integrates information from the Kyoto Encyclopedia of Genes and Genomes (KEGG) database, Human Metabolome Database (HMDB) and Human Protein Atlas (HPA).

**Methods and results:**

Existing literature was used to determine the roles of *CPS1* and *CAD* in HCC. *CPS1* downregulation is thought to play a role in hepatocarcinogenesis through an increased glutamine availability for de novo pyrimidine biosynthesis, which CAD catalyzes the first three steps for. KEGG, HMDB and HPA were used to reconstruct a network of relevant pathways, demonstrating the relationships between genes and metabolites using the MetaboSignal package in R. The network was filtered to exclude any duplicates, and those greater than three steps away from *CPS1* or *CAD*. Consequently, a network of 18 metabolites, 28 metabolic genes and 1 signaling gene was obtained, which indicated expression profiles and prognostic information of each gene in the network.

**Conclusion:**

Information from different databases was collated to form an informative network that integrated different “-omics” approaches, demonstrating the relationships between genetic and metabolic components of urea cycle and the de novo pyrimidine biosynthesis pathway. This study paves the way for further research by acting as a template to investigate the relationships between genes and metabolites, explore their potential roles in various diseases and aid the development of new screening and treatment methods through network reconstruction.

## Introduction

Hepatocellular carcinoma (HCC) is the most prevalent primary liver cancer. The latest GLOBOCAN data have estimated HCC to be the 4th biggest cause of global cancer-related mortality, accounting for 8.2% of all cancer-related deaths. Over 80% of HCC cases occur in sub-Saharan Africa and in Eastern Asia. However, HCC cases have also been increasing in low-incidence areas, such as the United States.^1^,^2^

Chronic liver inflammation of any underlying cause results in a cycle of necrosis and regeneration, leading to chromosomal instability and the accumulation of reactive oxygen species and inflammatory cytokines, resulting in genetic and epigenetic alterations.^3^–^5^ Although the main risk factors vary between regions, chronic hepatitis B infection and aflatoxin B1 exposure are key determinants in high-risk areas.^1^–^3^ The increasing prevalence of obesity is thought to contribute to the increasing HCC incidence in low-risk areas.^1^

The development of HCC is a multi-step process involving genetic and environmental interactions. Amongst the 26 genes identified recently to be frequently mutated in HCC by The Cancer Genome Atlas (TCGA) Research Network, several genes have been linked to metabolic reprogramming, such as the *APOB, CTNNB1* and *CPS1* genes.^6^

Carbamoyl Phosphate Synthetase I (*CPS1*) is an enzyme found in the mitochondrion, responsible for catalyzing the first and rate-limiting step of the hepatic urea cycle, critical for the removal of excess urea from cells.^7^,^8^ There is evidence to suggest that *CPS1* downregulation may play a critical role in the development of HCC in 5.92% of liver cancer cases according to TCGA.^6^,^9^,^10^

Networks are bioinformatic tools that are increasingly used to visualize relationships within biological systems, such as those between genes and their products.^11^ MetaboSignal (http://bioconductor.org/packages/release/bioc/html/MetaboSignal.html) is an R package which allows the development of networks that demonstrate the relationships between genes and metabolites through utilizing the information available from the Kyoto Encyclopaedia of Genes and Genomes (KEGG) database.^12^,^13^ In addition, information from other databases such as the Human Protein Atlas (HPA) and the Human Metabolome Database (HMDB) can be merged for the first time to be demonstrated in a single informative network that integrates various “-omics” approaches.^6^,^14^

This study aimed to identify the evidence for *CPS1* and c*arbamoyl phosphate synthetase/aspartate transcarbamoylase/dihydroorotase (CAD)* dysregulation in HCC based on existing literature and explore their metabolic impact through the reconstruction of a network that integrates information from the KEGG, HPA and HMDB databases.

## Methods

### Literature Review

A literature search was conducted to explore the mechanism behind the metabolic roles of both *CPS1 and CAD*, both in normal metabolism and in HCC. This was limited to articles in English. Additional information available from the European Molecular Biology Laboratory, HMDB and KEGG databases regarding *CPS1* and *CAD* was also obtained.

### Reconstruction of MetaboSignal Network

MetaboSignal is an R (R Development Core Team, Auckland, New Zealand) package that utilizes the KEGG database to reconstruct comprehensive networks exploring relationships between genes and metabolites. In order to reconstruct the network, the KEGG database was used to identify the metabolic pathways in which *CPS1* and *CAD* are involved, resulting in selecting the pyrimidine metabolism, arginine biosynthesis and alanine, aspartate and glutamate metabolism pathways, which were then merged, and any duplicates were removed. Distances of each metabolite node from *CPS1* and *CAD* were calculated and those that were greater than three steps away were disregarded for simplification purposes. This ability was granted through a MetaboSignal function, although since there was no evident use of this function in published data, the maximum number of steps was determined through trial and error, as any lower number of steps oversimplified the network, while higher resulted in networks that were complicated, resulting in networks that would not allow for any useful inferences. A node table (a list of all metabolites and genes in the network) and an edge table (a list of all connections between the genes and the metabolites i.e. the nodes) were created. Data which indicated the expression profiles of genes within the network in hepatocytes, and provided prognostic information based on patient survival and mRNA level correlation based on data from TCGA, HPA version 19 and Ensembl version 92.38 were incorporated into the nodes table. This data was obtained directly from tissue samples.^15^,^16^ Genes indicative of prognosis were identified as those with log rank p values less than 0.001 in Kaplan-Meier analysis. Those with a significantly lower number of overall survival than expected were labelled as “unfavorable” and vice versa. Data from HMDB were used to annotate whether each metabolite that was included in the network could be detected in normal and abnormal urine. The R script used to achieve these steps can be found in Supplementary Material I.

Cytoscape v3.7.0 (Institute for Systems Biology, Seattle, WA, USA) was used to visualize the network produced. The nodes represent each unit within the network (i.e. the metabolites and the genes), and were visualized as points. The edges, visualized as lines, represent the interactions between each node. The node table was used to manually filter out the genes that were not expressed in hepatocytes. Consequently, any nodes that were no longer connected to the network, and any gene nodes that were not connected to a minimum of two metabolic nodes were also filtered out as genes only connected to a single metabolite would not represent a complete reaction.

## Results

### Evidence for the Role of *CPS1* and *CAD* in HCC

**Figure 1:**
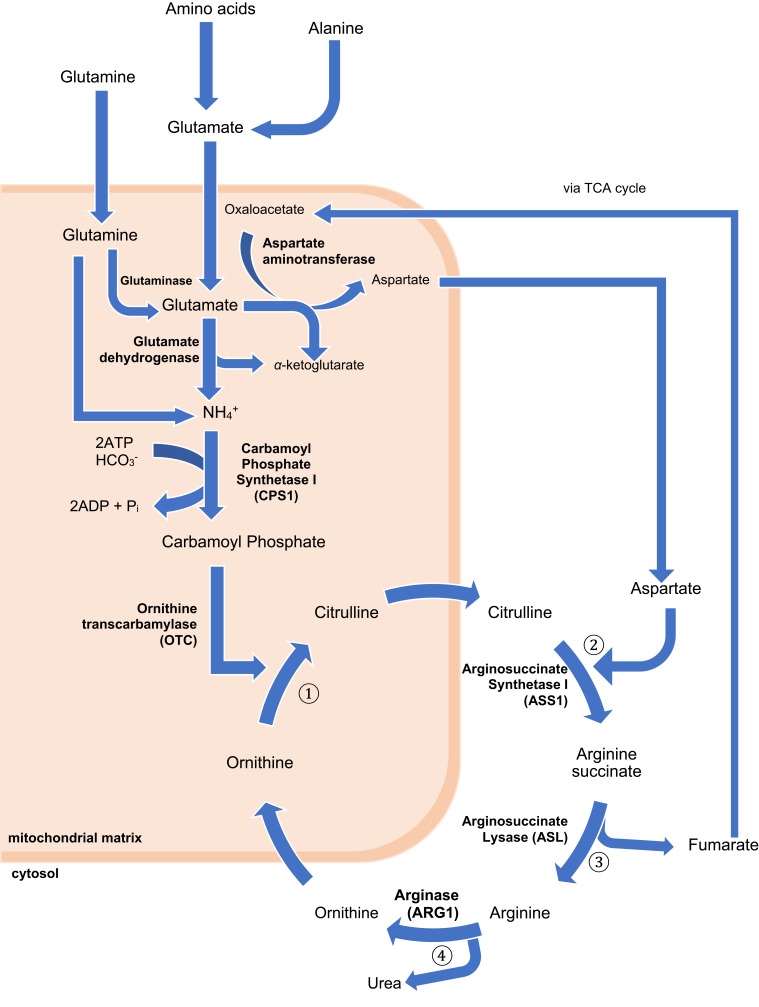
The urea cycle. The process begins in the liver mitochondrial matrix although some steps take place in the cytoplasm. During the metabolic breakdown of amino acids, most commonly, the cleaved amino group is transferred to α-ketoglutarate to form glutamate. Ammonia formed elsewhere can be transported to the liver to partake in the urea cycle in two ways; either as the amino group of alanine, if from skeletal muscle, or the amide nitrogen of glutamine, if formed in other tissues. Regardless of the source, all NH4+ generated within the mitochondria are used, together with CO2 and ATP to form carbamoyl phosphate, catalyzed by *CPS1*. This enters the urea cycle and initiates the four-step urea cycle. ① Ornithine and carbamoyl phosphate form citrulline, which passes into the cytosol. This is catalyzed by ornithine transcarbamylase (OTC) ② Formation of arginine succinate from a citrulline-aspartate intermediate, as catalyzed by arginosuccinate synthetase I (ASS1). The aspartate used in this step is generated via the action of aspartate aminotransferase on glutamate in the mitochondrial matrix. ③ Formation of arginine from arginine succinate. This leads to the release of fumarate, which enters the citric acid cycle. ④ Arginine is converted to ornithine by arginase (ARG1), also generating urea. Ornithine generated in this process moves from the cytosol to the mitochondrial matrix, allowing the continuation of the cycle.^8^

*CPS1* is a liver-specific enzyme responsible for catalyzing the first step of the urea cycle, converting ammonium into carbamoyl phosphate.^9^ (Figure 1) The *CPS1* gene is located on 2q35 on chromosome 2 and consist of 38 exons.^17^,^18^

**Figure 2:**
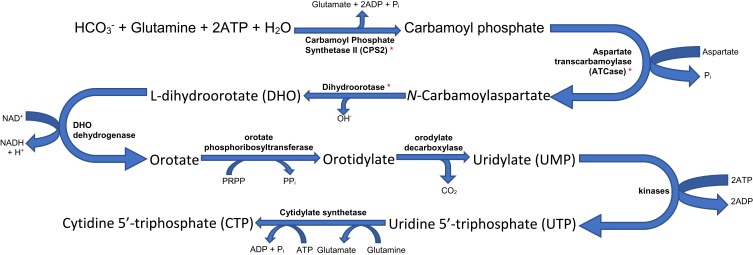
De novo pyrimidine biosynthesis pathway. Carbamoyl Phosphate Synthetase II (CPS2), the cytosolic form of the enzyme *CPS1* found in the mitochondrial matrix taking part in the urea cycle, catalyses the reaction leading to the formation of carbamoyl phosphate from HCO_3_^−^, glutamine, ATP and water. The generated carbamoyl phosphate and aspartate are used to generate pyrimidines though the steps as seen above. Note that thymidine 5ʹ-triphosphate (TTP), another pyrimidine is generated from CTP. Those marked * are components of the CAD complex.^8^,^25^,^26^

*CAD* encodes a trifunctional protein involved in the first three steps of de nov*o* pyrimidine synthesis (Figure 2). Carbamoyl phosphate synthetase II (CPS2), the first component of *CAD*, is the cytosolic isoform of *CPS1*, carrying out the rate-limiting step of this process.^6^,^19^

### Network Reconstruction

Upon merging the pyrimidine metabolism, arginine biosynthesis and alanine, aspartate and glutamate metabolism pathways, removing duplicates, and filtering out nodes more than 3 steps away from *CPS1* or *CAD*, a directed network of 71 nodes (20 metabolites, 47 metabolic genes and 4 signalling genes) and 138 edges (the connections between the genes and the metabolites) was obtained.

Following further filtering of genes not expressed in hepatocytes and nodes with a lack of connection to the network as described in 3.2, a final network with 47 nodes (19 metabolites, 27 metabolic genes and 1 signaling gene) and 74 edges was obtained (Figure 4) (Supplementary Material II), which successfully displayed the connection between the urea cycle and de novo pyrimidine synthesis, and thus *CPS1* and *CAD*.

The data from the TCGA and HPA which indicated expression profiles provided prognostic information was from 365 patients. (Table 1)^15^,^16^

**Table 1:** Table Summarizing Clinicopathological Information for the HPA Dataset

		Number of Participants	
Stage of HCC	1	170	
	2	84	
	3	83	
	4	4	
Missing stage information	24	
Gender		Male	Female
246	119
Total		365	

**Note:** Data from these studies.^15^,^16^

**Abbreviations:** HCC, Hepatocellular carcinoma; HPA, Human Protein Atlas.

Moreover, the network showed that an increased expression of *CPS1* was associated with a favorable prognosis (p=6.93x10^−5^), and an increased expression of *CAD* was associated with an unfavorable prognosis (p=1.72x10^−11^) based on data from HPA.

## Discussion

Metabonomics is the analysis of biological samples to determine composite biochemical profiles that reflect the metabolic status of an individual.^20^ Although in research “-omics” approaches are often investigated individually, they are intrinsically interlinked.^21^ This study was the first of its kind to develop a network displaying the interactions between genes and metabolites by using information from various databases. The network was used to investigate the role of *CPS1*, which codes for a protein that catalyzes the first step of the urea cycle, and *CAD*, which codes for a trifunctional protein catalyzing the first three steps of de novo pyrimidine biosynthesis pathway, in normal metabolism, and in turn, metabolic reprogramming in HCC.^7^,^8^,^22^–^25^

A downregulation of *CPS1* has frequently been associated with HCC in literature.^7^,^8^,^22^ The upregulation of *CAD* in HCC, which has been previously reported, was hypothesized to be linked to the downregulation in *CPS1* through an increased availability of glutamine due to the halting of urea cycle.^23^–^25^ This study aimed to develop a network which explored the relationships between genes and metabolites in disease, and to test the applicability of such networks, using *CPS1* and *CAD* in HCC as an example, and using information from KEGG, HMDB and HPA to develop the network.

### Identification of *CPS1* as Candidate Gene

Each gene that was identified to be a candidate for analysis for the purpose of this study has been identified to be involved in metabolic reprogramming in HCC.

Truncated mutations of *APOB* are speculated to result in decreased very low-density lipoprotein synthesis, diverting energy into cancer-related metabolic pathways.^27^,^28^ Activation of *CTNNB1* is thought to rewire fatty acid catabolism, resulting in reduced lipogenesis and increased use of fatty acids as an energy source.^29^,^30^ Silencing of *CPS1* in HCC is linked to an in increased *CAD* expression, leading to increased pyrimidine synthesis, allowing for increased cell proliferation.^6^

The choice of investigating *CPS1* for the purpose of this research was made upon considering several factors. Most importantly, literature review provided sufficient evidence that *CPS1* is commonly downregulated in HCC, with TCGA reporting *CPS1* abnormalities in 5.92% of liver cancer cases.^6^ A study theorized that the downregulation of *CPS1* in HCC may be due to the loss of differentiation in hepatocytes, but this was excluded, since other liver-specific genes were found to be adequately expressed.^9^

A 2011 study by Liu et al has found that instead of traditional CpG islands, the *CPS1* gene possesses two CpG dinucleotides. The hypermethylation of these dinucleotides are closely linked to the downregulation of *CPS1* in HCC. The same study was able to conclude that *CPS1* was under-expressed in cirrhotic liver tissue compared to healthy samples. Liu et al therefore concluded that *CPS1* methylation may be a suitable early biomarker for HCC.^9^

**Figure 3:**
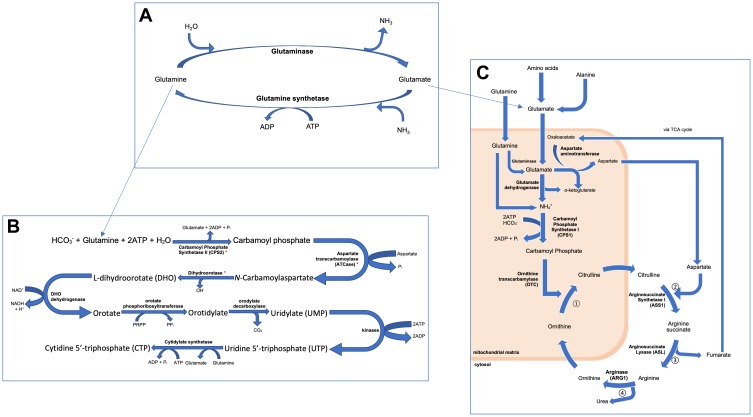
Figure summarising the role of (**A**) glutamine and glutamate synthesis, in (**B**) de novo pyrimidine biosynthesis and (**C**) the urea cycle. These pathways are thought to be intrinsically linked, as demonstrated in this figure. Consequentially, it was hypothesised that a downregulation in *CPS1* in HCC would result in an increased availability of glutamine for the biosynthesis of pyrimidines, favouring cell division, therefore promoting the development of HCC. Those marked with * in (**B**) are the individual components of the CAD complex, which are thought to be upregulated in HCC.

The downregulation of *CPS1* has been found to halt the urea cycle, diverting the amino acids involved in this process (glutamine, and glutamate, which can be converted to glutamine by glutamine synthetase) to initiate de novo pyrimidine synthesis through the action of the trifunctional protein coded by *CAD*, which may be upregulated in *CPS1*-silenced-HCC (Figure 3).^6^,^23^–^25^ TCGA has reported a 2.8-fold increase and a 2.1-fold decrease in *CAD* and *CPS1* RNA levels in HCC compared to normal hepatocytes respectively, with *CAD* abnormalities reported in 3.50% of liver cancers.^6^

Despite the evidence supporting the involvement of *CPS1* in HCC, there appears to be a gap in the understanding of the mechanism behind this process. Additionally, the other identified potential candidate genes such as *APOB* and *CTNNB1* are involved in signaling pathways, activation and/or deactivating other genes, while *CPS1* is directly involved in metabolism, thus is less prone to potential confounding due to the involvement of other genes within signaling pathways.^28^,^26^

### Network Filtering

It is important to note that the final network has undergone various steps of filtering, most notably to only include metabolites at most three steps from *CPS1* and *CAD*. Although this step has allowed for the much-needed simplification of the network, it has also filtered out a considerable number of nodes that may have been relevant to the study. While it can be argued that a more concise network can be easier to comprehend and thus be more informative, and that the further away from the target genes, the more likely that the concentrations of metabolites would be more prone to confounding, various nodes directly involved in the urea cycle, and more notably, in de novo pyrimidine synthesis (only the first 4 steps of the 8 step process demonstrated in Figure 3 can be located in the network) have been removed as a result of the filtering process.

### *CPS1, CAD* and Prognosis

The data integrated from HPA allowed the network to display how an increased expression of the genes influenced the prognosis of patients.^14^–^16^ Interestingly, an increased expression of *CPS1* was linked with a significantly more favorable prognosis (p=6.93x10^−5^) whereas an increased expression of CAD was linked with a significantly less favorable prognosis (p=1.72x10^−11^).

Both *CPS1* and *CAD* have been well established to play important roles in the development of HCC, mainly due to their relation to de novo pyrimidine synthesis.^8^,^22^–^25^ Although the same mechanism could also be linked to the effects of increased expression of *CPS1* and *CAD* on prognosis, by speeding up and slowing down cell proliferation respectively, no work has been done to investigate whether this is the case.

**Figure 4:**
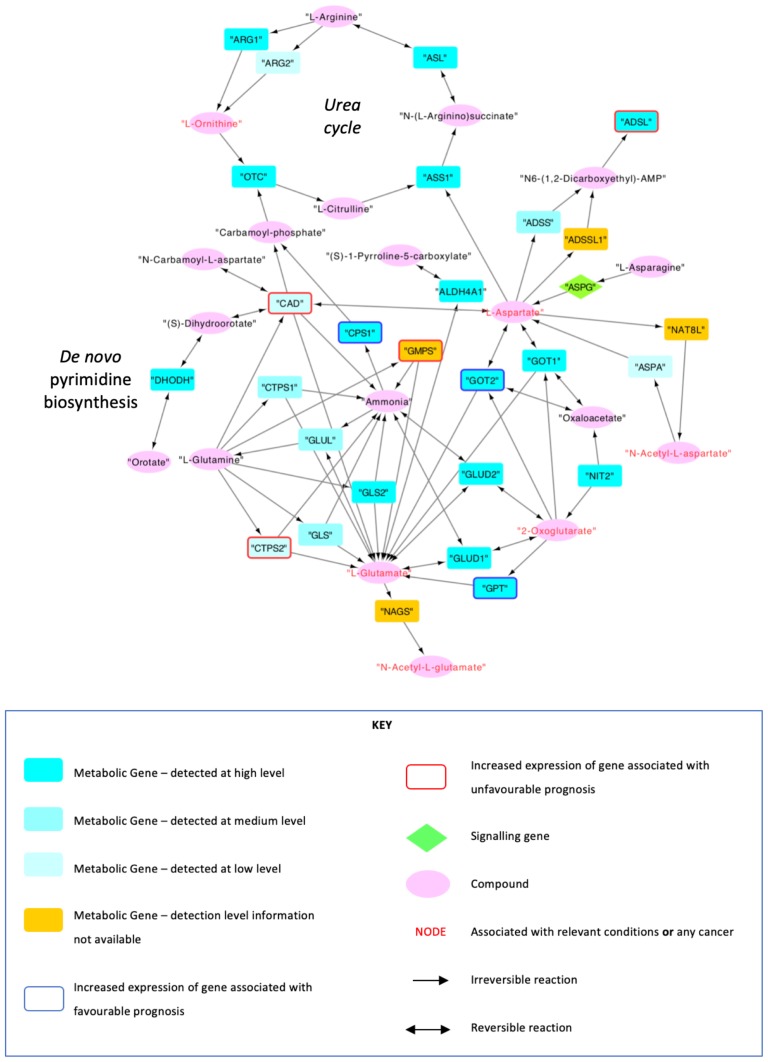
Network that was formed using data from KEGG, HPA and HMDB. The nodes were filtered to be at a maximum distance of 3 steps from *CPS1* and CAD, to show metabolites that are expressed in hepatocytes, and to genes that are connected to at least two metabolites. Data from TCGA and HPA were used to indicate whether any nodes were associated with any relevant conditions and cancers. The genes that were associated with favourable and unfavourable prognoses upon upregulation at an RNA level were also labelled using data from TCGA and HPA.

Although information regarding each individual pathway is freely available on KEGG, this is the first study to not only combine these several pathways using information from KEGG to form a network such as the one demonstrated in Figure 4, but also incorporate information from different databases such as HPA and HMDB, allowing for a comprehensive and informative approach regarding prognosis, disease associations and detection concentrations. However, the network was filtered to only include genes expressed in normal hepatocytes, which may differ to those expressed in HCC. This may have potentially resulted in metabolites relevant in HCC to be filtered out due to their lack of expression in healthy hepatocytes.

Future confirmatory work should extend this study into case-control analyses by validating the findings of the network through a metabonomic approach, investigating the metabolites within the network. This can be achieved by obtaining urine, serum and tissue samples from healthy volunteers, a cirrhosis cohort with a HCC cohort, and comparing the concentrations of the metabolites in the network that were found within the samples following a rigorous metabolite annotation process, which would allow to draw conclusions regarding the selected genes’ and the metabolites’ roles in HCC.

Alpha-fetoprotein (AFP) is the most widely available tumor biomarker used in the detection of HCC. However, a serious limitation of AFP in its diagnostic use is its unsatisfactory sensitivity and specificity at the designated cut-off value of 20 ng/mL, resulting in a need to utilize more invasive or radiological diagnostic methods to confirm the presence of HCC. Therefore, there is an urgent unmet need for a specific and sensitive biomarker in HCC, which can potentially be met through extending this study as noted.^31^

70–90% of HCC occurs on the background of chronic liver disease and cirrhosis, which is associated with different etiologies, such as chronic hepatitis B and hepatitis C infections, excessive consumption of alcohol, exposure to aflatoxin B1 and.^2^,^32^ Although research has been conducted to link specific mutations to individual etiologies in HCC, the extent of the findings from such research has been minimal.^33^ Methodology established by this study can be extended to determine whether the concentrations of any metabolite from the network differed in any underlying etiologies of HCC, which could allow conclusions to be reached regarding the role of specific genes such as *CPS1* and *CAD* in different etiologies of HCC.

## Conclusion

In summary, this study is the first to compile information from various databases such as HMDB, KEGG and HPA to form an informative network. The metabolic consequences of *CPS1* and *CAD* alterations were investigated through the construction of a network combining urea cycle and the de novo pyrimidine biosynthesis pathways, two of the many metabolic pathways which are thought to play a role in the development of HCC. Through the development of a modifiable R script, the study paved the way for further research to be able to combine information from different databases further investigate the relationships between genes and metabolites. There is currently an unmet need for specific and sensitive biomarkers for screening for HCC. This study can not only act as a template to fill this gap by aiding in the development of new techniques, potentially non-invasively via urine samples, to screen for HCC and other conditions.

## Abbreviations

HCC, Hepatocellular carcinoma; *CPS1*, Carbamoyl Phosphate Synthase I; KEGG, Kyoto Encyclopedia of Genes and Genomes; HMDB,Human Metabolome Database; HPA, Human Protein Atlas; CAD, Carbamoyl phosphate synthetase/aspartate transcarbamoylase/dihydroorotase; TCGA, The Cancer Genome Atlas; CPS2, Carbamoyl phosphate synthetase II.
